# Feature binding contributions to effect monitoring

**DOI:** 10.3758/s13414-020-02036-9

**Published:** 2020-04-28

**Authors:** Robert Wirth, Wilfried Kunde

**Affiliations:** Department of Psychology, Julius Maximilians University of Würzburg, Röntgenring 11, 97070 Würzburg, Germany

**Keywords:** Action control, Ideomotor theory, Action effects, Psychological refractory period, Effect monitoring

## Abstract

Monitoring the perceptual effects of body movements is supposed to be a capacity-limited process that can interfere with processing of a concurrent task. Here we studied the contribution of feature binding to such effect monitoring interference. In three experiments, we varied the possibility of feature overlap between responses and effects in a primary task and responses in a secondary task. We show that responses in a secondary task are delayed when they partially, rather than completely, alternate or repeat features of responses/effects of a primary task. Yet, these partial feature repetition/alternation costs are small, and they occur on top of other factors that lengthen the critical effect monitoring process, such as the spatial compatibility of responses and effects in the primary task. The results thus show that feature binding contributes to, but cannot fully account for, delays in a secondary task caused by monitoring effects of a primary task.

## Introduction

### Effect monitoring

Humans monitor what they do, and they do so for multiple reasons. First, actions are usually carried out with a certain outcome in mind, and it is thus important to know whether an action has produced the intended outcome or not. Second, even when action outcomes are not predictable (e.g., during exploration), it is important to constantly monitor whether specific contingencies between actions and outcomes exist, which then render these outcomes predictable, and therefore intentionally reproducible in the future. Take, for example, a newborn that cannot yet behave in a goal-directed and intentional manner. Instead, all the baby’s actions are involuntary, but still they produce sensory consequences, as, for example, the specific proprioceptive feeling that comes with moving the right arm, or the sound that arises when straining the vocal cords, or the visual changes from moving the head in one direction. Repeatedly experiencing what motor patterns produce which perceptual consequences (i.e., action effects) establishes a link between these motor patterns and their effects. Monitoring is thus a perceptual process that allows for recognizing these contingencies, which is a necessary prerequisite for intentional behavior. As with the examples above, irrespective of whether perceptual consequences of motor output originate from the own body (e.g., the feeling of moving an arm) or from the environment (e.g., the sound of a rattle held in a moving arm), any reliable contingency between a motor pattern and its perceptual outcome can be the basis for goal-directed behavior (Pfister, 2019; Wirth, Brandes, Pfister, & Kunde, 2016).

**Fig. 1:**
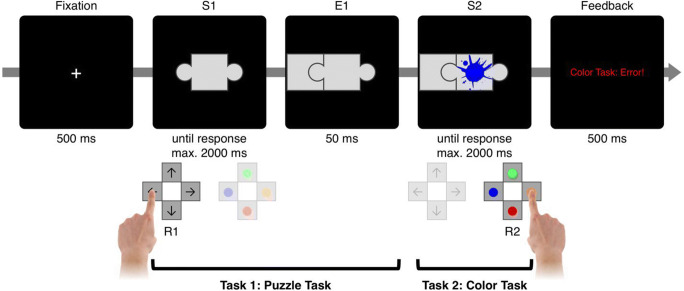
Procedure of the experiments. After a fixation cross, the stimulus for Task 1 (S1) appeared and called for the production of a puzzle piece at the location of a connector (E1), via keypress (R1). After a delay of 50 ms, a color splash was presented (S2) and had to be categorized via another keypress (R2). Feedback was provided only in case of errors or omissions, otherwise a new trial started immediately

The idea of effect monitoring had been envisioned early on by Welford (1952). Welford discussed this process in the context of dual tasking, and he assumed that such monitoring does interfere with concurrent tasks when he proposed that “*the perception of any such feedback will require central organizing time*” (p. 18). Yet, until recently, empirical evidence for the assumed capacity limitation of the effect monitoring process was scarce. There is research showing that humans monitor their motor responses, which likely entails monitoring of the proprioceptive and/or visual feedback of body movements. Such response monitoring can in fact delay processing of a temporally overlapping task, supporting the capacity-limited character of such monitoring (Bratzke, Rolke, & Ulrich, 2009; Jentzsch, Leuthold, & Ulrich, 2007; Ulrich et al., 2006). However, these studies mainly relied on continuous motor responses, such as moving a handle over a shorter or longer distance. This makes it difficult to disentangle the pure contribution of monitoring the movement effects from the process of generating the movement in the first place. To better distinguish the process of generating a response from the process of monitoring its perceptual effects, we suggested a different approach, where motoric responses are kept constant, but only the perceptual effects of these responses vary, which we describe in more detail (Wirth, Janczyk, & Kunde, 2018a, Experiment 1; see Fig. 1 for a similar setup):

For Task 1, participants were confronted with a puzzle piece on screen that had connectors at the left, at the right, or at both sides (S1), and participants had to add a puzzle piece via the press of a left or a right button wherever it fit (R1). As soon as a button was pressed, the corresponding puzzle piece appeared on-screen as an effect of that button press (E1). Crucially, the relation between the button press and the location of the puzzle piece on-screen was manipulated. In some blocks, they were related in a spatially compatible manner, meaning button presses would produce puzzle pieces on the same side (R1→E1: left→left; right→right), whereas in other blocks, they were related in an incompatible manner (left→right; right→left). Previous research suggests that the production of an effect that would predictably be spatially compatible should be easier (i.e., quicker response times (RTs) and less errors) than the production of a predictably incompatible effect (e.g., Kunde, Pfister, & Janczyk, 2012). More importantly, a similar logic was applied to effect monitoring, assuming that monitoring spatially compatible effects might be completed faster than monitoring spatially incompatible effects (speculatively because they might be encoded and processed more easily, e.g., Hommel & Schneider, 2002; Müsseler, Wühr, Danielmeier, & Zysset, 2005). If this were the case (and if effect monitoring drew on cognitive resources; Welford, 1952), then a second task that would have to run in parallel to the effect monitoring process should be delayed by the still ongoing monitoring in the first task. To test that, for Task 2, one of two color patches appeared on screen (S2), 50 ms after the puzzle piece had been produced. Participants were to categorize the color via button-press (R2).

The results consistently showed that responses in Task 2 were significantly slower after an R1-E1 incompatible effect compared to after a compatible effect.^1^ This can be explained by assuming that monitoring incompatible effects takes longer than monitoring compatible effects. With longer monitoring after Task 1 (i.e., after incompatible effects), there is a longer temporal overlap between the monitoring process and the processing of Task 2. This longer overlap comes with a higher chance of interference between the two processes, and consequently, longer RTs after incompatible effects. This result has since been replicated and elaborated on several times (e.g., Kunde, Wirth, & Janczyk, 2018; Steinhauser, Wirth, Kunde, Janczyk, & Steinhauser, 2018; Wirth, Janczyk, & Kunde, 2018a; Wirth, Steinhauser, Janczyk, Steinhauser, & Kunde, 2018b). Among other things, we have demonstrated that this effect occurs with blocked and trial-wise manipulations of R1-E1 compatibility, ruling out most strategic explanations (Wirth, Janczyk, & Kunde, 2018a, Experiments 5a and 5b; results also replicated here in Experiment 2). Also, the same effect occurs when there is no response-specific S1, ruling out possible explanations in terms S1-E1 compatibility.

To summarize, empirical data now suggest that we monitor the consequences of our actions, and that certain factors, such as the spatial compatibility between responses and effects (among other factors), can lengthen or shorten this monitoring process. During such monitoring, another concurrent task cannot run with the same efficiency as without such monitoring taking place, and the higher the monitoring affordances, the slower are RTs in the concurrent task.

Based on these results, we want to further elaborate on what exactly renders effect monitoring as a process to interfere with a concurrent or subsequent task. However, we do not want to propose that there is one general answer to this question, as the exact attentional and cognitive processes that are involved in effect monitoring may vary wildly between tasks. But with spatial effect compatibility, as described above, feature binding processes that are assumed to take place when two (or more) perceptual or motor events co-occur, might represent a stimulus-driven, hard-to-overcome limitation of our cognitive system (Hommel, 1998, 2004).

### Feature binding

Feature binding assumes that when responding to a stimulus, binary bindings are formed between the stimulus and response features, and repeating any of the integrated features shortly after would automatically retrieve the whole episode. If in a sequence both stimuli and responses repeat, responses are fast, as the whole event file can be recycled (full repetitions). Similarly, if in a sequence both stimuli and responses alternate, responses are fast, as none of the currently required features are pre-bound and can be linked easily (full alternations). Performance costs arise when only stimuli repeat and responses alternate, or vice versa (partial alternations), because the repeated features (a) retrieve still bound, but currently not needed features, and (b) have to be “unbound” before they can be integrated into a new event file (e.g., Kahneman, Treisman, & Gibbs, 1992; Hommel, 1998, 2004). In their original formulation, event files consisted of only stimuli and responses. However, recent work has very consistently demonstrated that event files can also contain other information, such as sensory consequences of a given response (e.g., Dutzi & Hommel, 2009; Janczyk, Heinemann, & Pfister, 2012; Moeller, Pfister, Kunde, & Frings, 2016). Feature binding has been a prominent account to explain all sorts of psychological phenomena in recent years, as the framework can be applied broadly (e.g., Henson, Eckstein, Waszak, Frings, & Horner, 2014). Still, we want to emphasize that there are clear limitations to feature binding (e.g., Schöpper, Hilchey, Lappe, & Frings, 2020).

### Effect monitoring = feature binding?

If the production of an effect creates an event file including features^2^ of R1 and E1 (Frings et al., 2020; Moeller, Pfister, Kunde, & Frings, 2019), this binding will then interfere with responses in a subsequent task (for a more detailed look, see Table 1). If spatial actions produce spatially compatible effects in a primary task (left→left; right→right), then a second task that also requires a left or right button press (neglecting any effect that R2 may produce) might either repeat both features of the event file (if both tasks require the same spatial response; full feature repetition), or it might not require any of the previously bound features at all (if both tasks require different spatial responses; full feature alternation). If actions in the primary task produce spatially incompatible effects (left→right; right→left), then the same second task always has to repeat either the R1-code or the E1-code of the previous episode (partial feature alternation), but never both. A vast literature shows that repeating some but not all features of an event file leads to worse performance (e.g., slowed down responses in the second task) compared to repeating all or none of the previously bound features (partial alternation costs, e.g., Hommel, 2004).

**Table 1.**
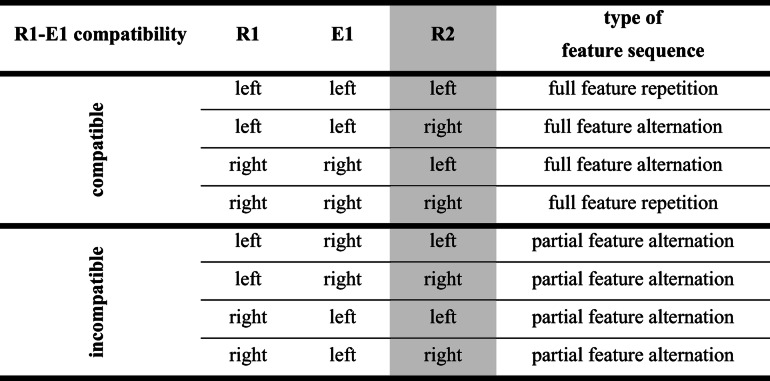
Full/partial repetitions/alternations of R2 features and R1-E1 features

Assuming the perspective of feature binding, the mechanism of binding R1 and E1 features in Task 1 and repeating all, none, or only some of these bound features in Task 2 might suffice to explain the slowdown after incompatible compared to after compatible action effects that we previously attributed to effect monitoring. In our previous setups, effect monitoring contributions and feature binding contributions are inherently confounded (see Table 1). With the current experiments, we will disentangle their contribution to the slowdown in a subsequent task, to check whether the limitation of feature binding can fully explain RT costs of monitoring incompatible rather than compatible action effects.

## Experiment 1

To test the potential feature binding contributions to effect monitoring, we designed an experiment in which we varied the response dimensions in Task 1 and Task 2 (horizontal vs. vertical) so that R2 feature repetitions were either possible (when relying on the same spatial dimension) or impossible (when relying on different spatial dimensions). In Task 1, participants produced effects that were either spatially compatible or incompatible, and Task 2 required them to categorize colors.

If both tasks rely on the same response dimension (both horizontal, as in Table 1, or both vertical), a compatible action effect in Task 1 would always lead to a full repetition or a full alternation in Task 2, and an incompatible action effect in Task 1 would always lead to a partial feature alternation (and, hence, slowdown) in Task 2. But if both tasks relied on different response dimensions, in no case would R2 repeat any of the spatial features that were bound in Task 1, leading to full alternations in both compatible and incompatible trials. Therefore, if a slowdown in Task 2 after an incompatible action effect is only found with dimensional overlap between the two tasks, but strongly reduced or even absent without dimensional overlap, then these incompatibility costs mainly constitute feature binding limitations. However, if a Task 2 slowdown after an incompatible effect is found irrespective of the dimensional overlap of the two tasks, then feature binding only plays a minor role in monitoring (spatially compatible and incompatible) effects.

### Method

#### Participants

Forty-eight participants were recruited (mean age 25.6 years, SD 4.4) and received monetary compensation. This sample size was chosen because it was the minimum number of participants required for counterbalancing, and it should provide high statistical power (>.99) for the critical propagation of the compatibility effect onto Task 2, assuming similar effect sizes to those previously found (Wirth, Janczyk, & Kunde, 2018a, Experiment 1). All participants reported normal vision and were naïve concerning the hypotheses of the experiment. All participants provided written informed consent prior to the experiment. One participant was removed from the final sample due to performing below chance level and was replaced.

#### Apparatus and stimuli

All stimuli were presented on a 22-in. screen against a black background (see Fig. 1). S1 were pictures of puzzle pieces with a connector at the left, right, top, bottom, or at two opposing sides (left and right, or top and bottom), presented centrally on the screen. If the puzzle piece included only one connector, a puzzle piece had to be produced that fitted the connector (forced choice). If two connectors were presented, participants could choose freely at which side they wanted to add a piece (free choice). In free-choice trials, participants were encouraged to choose their option spontaneously while maintaining an approximately equal ratio between all options. Participants responded with the left hand on the “S”, “D”, “E”, or “X” key of a standard QWERTZ keyboard (R1). These keypresses produced puzzle pieces of the screen as an effect (E1).

For Task 2, participants had to categorize a splash according to its color (S2). The color splash was presented centrally within S1 and required a response with the right hand on the “2”, “4”, “6”, or “8” key on the number pad (R2).

#### Procedure

The trial procedure is illustrated in Fig. 1. A fixation cross marked the beginning of a trial. After 500 ms, S1 was presented centrally on the screen and required the production of E1 via pressing a key with the left hand (R1). The produced puzzle piece appeared immediately after R1 and stayed on-screen until the trial ended. If, after a maximum of 2,000 ms, no R1 key was pressed, this counted as an R1 omission and no E1 was displayed.

After an interval of 50 ms after E1 onset (or after 2,050 ms after S1 onset in case of R1 omissions), S2 was displayed centrally and called for R2. Again, if no key was pressed within 2,000 ms, this counted as an R2 omission. The two tasks were always presented in that order, and there was no temporal overlap between the tasks (except for the effect monitoring process). In case of any errors or omissions, written feedback was presented at the end of the trial (e.g., “Puzzle task: Too slow!”, or “Color task: Error!”, or both) for 500 ms in red. If both tasks were completed correctly, the next trial, indicated by a fixation cross, started immediately.

**Fig. 2:**
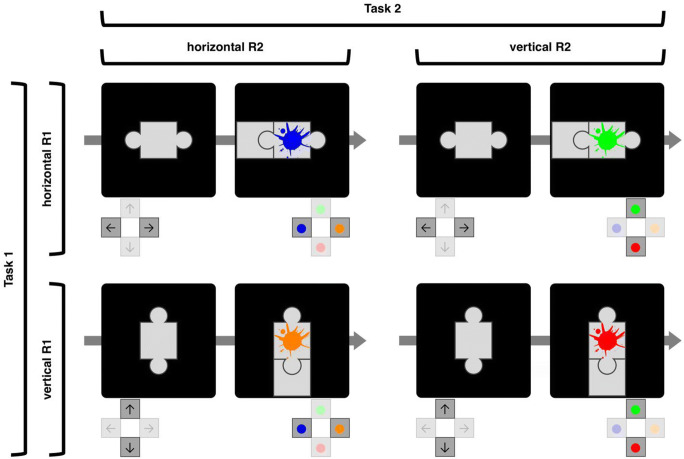
Illustration of R1-R2 combinations. Task 1 could either require horizontal responses, so that only puzzle pieces with left or right (or both) connectors would be presented (top row), or it could require vertical responses, with only top or bottom (or both) connectors (bottom row). Similarly, Task 2 could either require horizontal responses, so that only blue and orange splashes would appear (left column), or it could require vertical responses, with only red and green splashes (right column). Together, both tasks could require the same response dimension (dimensional overlap conditions: top left and bottom right illustrations) or different response dimensions (no dimensional overlap conditions: top right and bottom left illustrations)

Two factors were experimentally manipulated. First, the response dimensions for both tasks were varied between blocks (R1-R2 combinations, see Fig. 2). In half of the blocks, Task 1 required only horizontal R1s, so that only puzzle pieces with left and right connectors were presented. In the other half of the blocks, only vertical R1s were required, presenting only top or bottom connectors (“rows” in Fig. 2). Orthogonally to the R1 dimension, the response requirements in Task 2 were also varied. In half of the blocks, Task 2 also only required horizontal R2s, so that only blue and orange splashes were displayed. In the other half of the blocks, only vertical R2s were required, presenting only red and green splashes (“columns” in Fig. 2). The combination of both variations resulted in blocks that either used the same response dimension in both tasks (dimensional overlap conditions: horizontal R1 & R2; vertical R1 & R2), or different response dimensions for both tasks (no dimensional overlap conditions: vertical R1 & horizontal R2; horizontal R1 & vertical R2). Participants were informed before each block which response dimension would be relevant for both tasks during the upcoming block.

Second, the spatial compatibility between response and effect in Task 1 was varied between experimental sessions (R1-E1 compatibility). The R1-E1 mapping could either be spatially compatible, so that the relative location of R1 would match the location of the E1 that it would produce (R1→E1 combinations: left→left; right→right; top→top; bottom→bottom; see Fig. 1 for an example), but it could also be incompatible, with responses producing effects on the opposite side (left→right; right→left; top→bottom; bottom→top). R1-E1 compatibility was constant for a whole session, and participants would be reminded before each block about the current compatibility.

Participants completed two sessions that were scheduled on different days, with 20 blocks each, five blocks per R1-R2 combination (see Fig. 2). One session employed a compatible R1-E1 mapping throughout, the other employed the incompatible R1-E1 mapping. The order of the sessions (compatible first vs. incompatible first), as well as the order of the R1-R2 combinations within these sessions (24 possible sequences), was counterbalanced across participants. Each block consisted of 80 trials, with 40 forced-choice trials and 40 free-choice trials in randomized order. In forced-choice trials, each combination of S1 (left vs. right connector; or top vs. bottom connector; depending on the R1 dimension of the current block) and S2 (blue vs. orange splash; or red vs. green splash; depending on the R2 dimension of the current block) was presented ten times (2 × 2 × 10), and in free-choice trials, the free-choice stimulus (left and right connectors; or top and bottom connectors; depending on the R1 dimension of the current block) was paired with each S2 20 times (1 × 2 × 20).

### Results

In free-choice trials, all four response options in Task 1 were chosen with an equal ratio (left key: 24.9%, right key 25.0%, top key: 24.5%, bottom key 25.2%, all *t*s<1, response omissions: 0.4%). For RT analyses, we excluded trials with errors (Task 1: 6.5%, Task 2: 8.1%) and omissions (Task 1: 0.4%, Task 2: 0.3%). The remaining trials were screened for outliers and we removed trials in which RTs for any task deviated more than 2.5 standard deviations from the corresponding cell mean, computed separately for each participant and experimental condition (4.3%). Overall, 15.7% of the trials were removed.

**Fig. 3:**
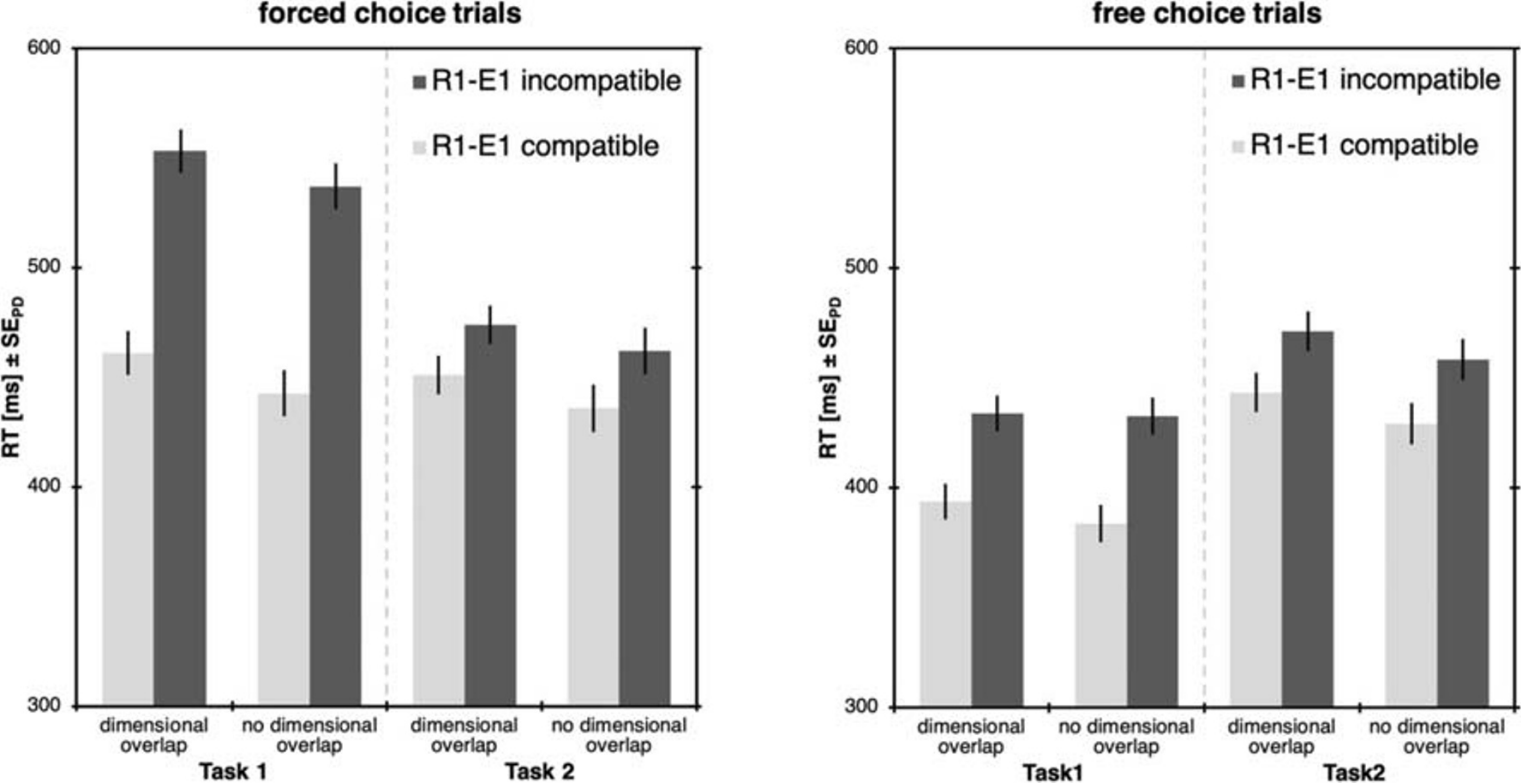
Results of Experiment 1. Response times (RTs) for forced-choice trials (**left**) and free-choice trials (**right**), separately for Task 1 and Task 2. Light bars represent trials with the spatially compatible R1-E1 mapping in Task 1, dark bars represent trials with the incompatible mapping. Error bars denote the standard error of paired differences, computed separately for each comparison of R1-E1 compatibility (Pfister & Janczyk, 2013)

The remaining data was then analyzed separately depending on whether Task 1 was forced or free choice. RTs were analyzed with a 2 × 2 ANOVA with Task 1 R1-E1 compatibility (compatible vs. incompatible) and type of R1-R2 dimensional overlap (overlap vs. no overlap) as within-subjects factors (see Fig. 3). Error rates and omissions were analyzed accordingly. As the interaction of compatibility and dimensional overlap in RT2s are the crucial result here, these interactions will be followed up via Bayes factor analysis.

#### Forced-choice trials

##### Task 1

In Task 1, we observed faster responses for the compatible R1-E1 mapping (452 ms) compared to the incompatible mapping (545 ms), *F*(1,47)=91.84, *p*<.001, η_p_^2^=.66. Further, there were faster responses in Task 1 when response dimensions differed between tasks (490 ms) than when they overlapped (507 ms), *F*(1,47)=7.63, *p*=.008, η_p_^2^=.14. There was no interaction between the two factors, *F*<1.

##### Task 2

The compatibility manipulation in Task 1 propagated to Task 2, *F*(1,47)=7.59, *p*=.008, η_p_^2^=.14, with faster responses after compatible effects (443 ms) compared to after incompatible effects (468 ms). Similarly, there were faster responses in Task 2 when response dimensions differed between tasks (449 ms) than when they overlapped (463 ms), *F*(1,47)=9.51, *p*=.003, η_p_^2^=.17. There was no interaction between the two factors, *F*<1, BF_01_=5.60, indicating that compatibility effects were of similar size both with, *t*(47)=2.76, *p*=.008, *d*=0.40, ∆=18 ms, and without dimensional overlap, *t*(47)=3.18, *p*=.003, *d*=0.46, ∆=20 ms.

##### Errors

Errors were more prominent in Task 1 with the incompatible mapping (6.8%) compared to the compatible mapping (3.7%), *F*(1,47)=27.14, *p*<.001, η_p_^2^=.37. The main effect of dimensional overlap was not significant, *F*<1. An interaction, *F*(1,47)=4.20, *p*=.046, η_p_^2^=.08, indicated more errors with the incompatible mapping relative to the compatible mapping in the dimensional overlap condition (∆=3.4%), but not without dimensional overlap (∆=2.7%). Errors in Task 2 did not show any significant effects, *F*s≤2.16, *p*s≥.148.

##### Omissions

Omission rates in Task 1 did not show any main effects, *F*s≤1, *p*s≥.326, but they were more prominent with the incompatible mapping relative to the compatible mapping in the dimensional overlap condition (∆=0.2%), but not without dimensional overlap (∆=0.0%), *F*(1,47)=4.12, *p*=.048, η_p_^2^=.08. Omission rates in Task 2 did not show any significant effects, *F*s≤3.57, *p*s≥.065.

#### Free-choice trials

##### Task 1

In Task 1, we observed faster responses for the compatible mapping (389 ms) compared to the incompatible mapping (433 ms), *F*(1,47)=32.75, *p*<.001, η_p_^2^=.41. Neither the main effect of dimensional overlap, *F*(1,47)=1.60, *p*=.213, η_p_^2^=.03, nor the interaction, *F*(1,47)=2.27, *p*=.139, η_p_^2^=.05, reached significance.

##### Task 2

Importantly, the compatibility manipulation in Task 1 propagated to Task 2, *F*(1,47)=11.47, *p*=.001, η_p_^2^=.20, with faster responses after compatible effects (436 ms) compared to trials after incompatible effects (465 ms). Further, there were faster responses in Task 2 when response dimensions differed between tasks (444 ms) than when they overlapped (457 ms), *F*(1,47)=11.30, *p*=.002, η_p_^2^=.19.^3^ There was no interaction between the two factors, *F*<1, BF_01_=6.32, indicating that compatibility effects were of similar size both with, *t*(47)=3.34, *p*=.002, *d*=0.48, ∆=24 ms, and without dimensional overlap, *t*(47)=3.61, *p*=.001, *d*=0.52, ∆=23 ms.

##### Errors

Errors revealed neither main effect nor interaction in both Task 1, *F*s≤2.84, *p*s≥.099, and Task 2, *F*s<1.

##### Omissions

Omission rates did not show any significant effects in either Task 1, *F*s≤3.23, *p*s≥.079, or Task 2, *F*s≤2.07, *p*s≥.157.

### Discussion

In Experiment 1, we tested whether feature binding processes contribute to the monitoring of spatial action effects. Task 1 required participants to produce a foreseeably compatible or incompatible effect via keypress, and Task 2 had them categorize color splashes. Further, the response dimensions were manipulated, so that both tasks relied on the same spatial dimension (both vertical or both horizontal), allowing for partial feature alternation costs in Task 2, or they relied on different response dimensions (one vertical, one horizontal), which eliminates any feature overlap to Task 2.

As expected, we found that the production of an incompatible effect takes longer and is more prone to errors than the production of a compatible effect. As these effects are not present at the time that the response is given, this influence on response production must be based on anticipatory codes of the effects (e.g., Kunde, 2001, 2003; Kunde, Schmidts, Wirth, & Herbort, 2017; Pfister & Kunde, 2013; Pfister, Janczyk, Wirth, Dignath, & Kunde, 2014; Wirth, Pfister, Janczyk, & Kunde, 2015). Also, replicating our previous results, we showed that with a dimensional overlap between the two tasks, responses after incompatible effects took longer to complete than responses after compatible effects (Wirth, Janczyk, & Kunde, 2018a; Wirth, Steinhauser, Janczyk, Steinhauser, & Kunde, 2018b). This slowdown after incompatible compared to after compatible action effects denotes increased costs of monitoring, as Task 2 itself did not employ any compatibility manipulation, and the still ongoing monitoring is the only cognitive process that could propagate to Task 2. This was not only true for forced-choice trials, but crucially also for free-choice trials that are free from potential influences of S-R compatibility.

Up until now, we have explained these response costs after incompatible trials exclusively as an indication for longer effect monitoring processes, interfering with the processing of the second task (e.g., Kunde, Wirth, & Janczyk, 2018); however, in this setup, these costs might also constitute partial alternation costs (see Table 1). Therefore, the crucial test to show costs for monitoring self-produced effects is when feature binding cannot contribute to the effect monitoring process, which is the case if the response dimensions of the two tasks do not overlap. In this condition we still find response costs after incompatible effects relative to after compatible effects, demonstrating that feature binding at best has a minimal influence on the effect monitoring process. That said, we do find an overall benefit for Task 2 if response dimensions differed between tasks in RTs, so our setup is in principle able to detect feature binding influences, but the limitations of binding events into event files seem to be independent of the limitations that occur when monitoring self-produced action effects.

Please note that participants used different hands for the two tasks, so there was a hand switch in each trial. This may have lowered our chances to find feature binding contributions in the first place, as participants did not repeat the identical responses (e.g., with their left index finger), but rather just the abstract spatial code of the response (e.g., “right”). We intentionally designed our experiment this way to disentangle feature binding contributions from motor affordances; however, this way we may somewhat underestimate the main effect of feature binding.

What is also noteworthy is that S2 was always presented 50 ms after R1 was given, so in fact, participants also produced the S2 onset on screen, so that S2 might also be construed as an action effect and might be integrated into the event file of Task 1. This might be the case, and we have no hard evidence against this, but several points make this unlikely. First, only the time of S2 onset was linked to R1, whereas the identity of S2 was completely unpredictable, making it less likely to be perceived as action-contingent against the perfectly controllable puzzle piece (but see Experiment 2 for unpredictable action effects). Second, we have previously found that monitoring costs also arise with a variable interval between the two tasks (Wirth, Janczyk, & Kunde, 2018a, Experiment 4). This does not directly speak against the idea that S2 is also integrated in the Task 1 event file, but it shows that the fixed time interval cannot drive the data pattern found in Task 2. Finally, even if S2 were integrated in the Task 1 event file as a secondary effect, S2 does not have an inherent spatial direction (as R1, E1, and R2), but only varies in color, so that we think it is unlikely that S2 could interfere with any of the results that we presented here.

To sum up, we found that monitoring costs in Task 2 due to an incompatible effect in Task 1 seems to be rather free from specific feature binding contributions. In the following two experiments, we will further elaborate on these results by (a) checking whether they hold in situations in which the spatial compatibility of R1 and E1 are less task relevant (Experiment 2), and (b) by dissecting what kind of feature overlap in incompatible trials (R1-R2 vs. E1-R2) mainly drives the slowdown in partial alternations relative to full repetitions/alternations (Experiment 3).

## Experiment 2

In Experiment 2, we wanted to replicate the results of Experiment 1, but address two potential alternative explanations. First, we saw that the compatibility manipulation in Task 1 was very strong, producing large effects, whereas the effects that were obtained with the manipulation of the response dimensions were far weaker (even though they were consistently observed). One might assume that the large compatibility effects could possibly obscure the minuscule influence that feature binding might potentially have on effect monitoring. Second, we manipulated spatial compatibility between sessions in Experiment 1, which allows for strategic or motivational influences. As described in the *Introduction*, if in a session a participant always produces incompatible effects, they might be more cautious overall to avoid errors, thereby increasing their overall RT, or they might also be less motivated, as they are consistently confronted with the more difficult task, similarly leading to slower responses.

To address both these potential confounds, in Experiment 2 we varied compatibility randomly from trial to trial. That way, participants are unpredictably confronted with both compatible and incompatible effects in the same block, eliminating any systematic strategic or motivational influences. Also, we should no longer find an effect of compatibility in Task 1 via effect anticipation, as they are no longer predictable, and the effect location no longer serves as a feedback signal on participants accuracy. Thereby we lessen the focus on the effects overall, and effect compatibility is no longer relevant for completing Task 1. With this setup, we can see how unpredictable effects are monitored (cf., Wirth, Janczyk, & Kunde, 2018a, Exp. 5), and whether feature binding contributes to effect monitoring under these circumstances.

### Method

Forty-eight new participants were recruited (mean age 25.0 years, SD 7.6) and received monetary compensation. They fulfilled the same criteria as in Experiment 1.

Apparatus, stimuli, and procedure were exactly as in Experiment 1 with the following changes: Now there was only one experimental session, and R1-E1 compatibility was varied randomly from trial to trial. That way, participants could not anticipate in advance whether they would produce a compatible or an incompatible effect. Therefore, only free-choice stimuli with two opposing connectors were used to visualize that two outcomes, a compatible E1 and an incompatible E1, were equally likely. Again, participants were informed before each block which response dimension would be relevant for both tasks during the upcoming block, but that R1-E1 compatibility could not be anticipated.

Participants again completed 20 blocks, five blocks per R1-R2 combination (see Fig. 2). Again, the order of the R1-R2 combinations was counterbalanced across subjects. Each block consisted of 80 trials, in which the free-choice stimulus (left and right connectors; or top and bottom connectors; depending on the R1 dimension of the current block) was paired with each S2 (blue vs. orange splash; or red vs. green splash; depending on the R2 dimension of the current block) and each R1-E1 mapping (compatible vs. incompatible) 20 times (1 × 2 × 2 × 20).

### Results

**Fig. 4:**
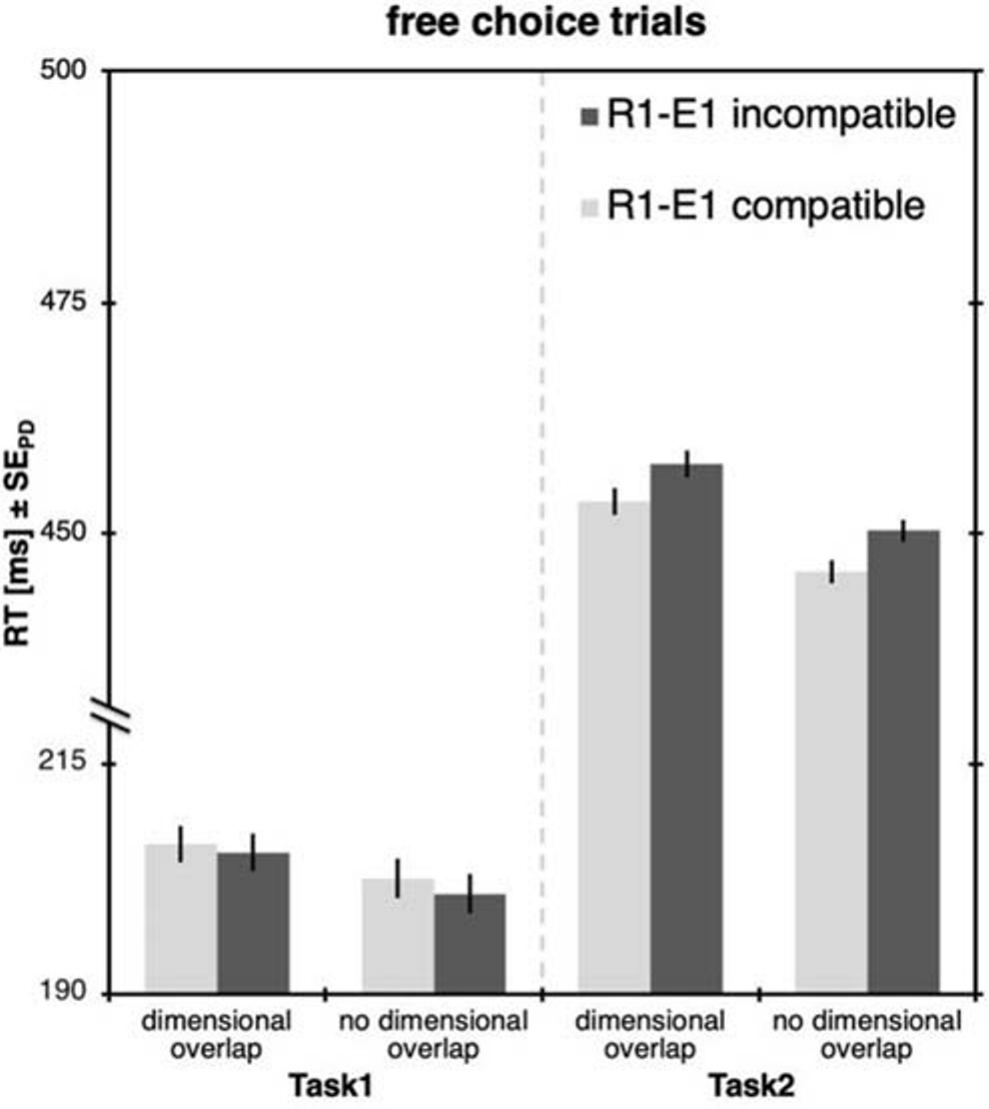
Results of Experiment 2. Response times (RTs) for Task 1 and Task 2. Light bars represent trials with the spatially compatible R1-E1 mapping in Task 1, dark bars represent trials with incompatible mapping. Error bars denote the standard error of paired differences, computed separately for each comparison of R1-E1 compatibility (Pfister & Janczyk, 2013)

All four response options in Task 1 were chosen with an equal ratio (left key: 24.6%, right key 25.2%, top key: 24.7%, bottom key 25.0%, all *t*s<1, response omissions: 0.4%). For RT analyses, we excluded trials with errors (Task 1: 0.2%, Task 2: 5.4%) and omissions (Task 1: 0.4%, Task 2: 0.2%). Outliers were removed as in Experiment 1 (4.9%). Overall, 11.0% of the trials were removed. The remaining data were then analyzed akin to the free-choice trials in Experiment 1 (see Fig. 4). Error rates were analyzed accordingly.

#### Task 1

Task 1 produced no significant results, *F*s<1.

#### Task 2

The compatibility manipulation in Task 1 showed up in Task 2, *F*(1,47)=17.21, *p*<.001, η_p_^2^=.27, with faster responses after trials with the compatible mapping (450 ms) compared to trials after the incompatible mapping (454 ms). Further, there were faster responses in Task 2 when dimensions differed between tasks (448 ms) than when they overlapped (455 ms), *F*(1,47)=4.40, *p*=.041, η_p_^2^=.09.^4^ There was no interaction between the two factors, *F*<1, BF_01_=1.13, indicating that compatibility effects were of similar size both with, *t*(47)=2.32, *p*=.025, *d*=0.33, ∆=2 ms, and without, *t*(47)=4.05, *p*<.001, *d*=0.58, ∆=4 ms, dimensional overlap.

#### Errors

Errors rates in Task 1 showed fewer errors for blocks with dimensional overlap (0.1%) relative to blocks without overlap (0.3%), *F*(1,47)=5.01, *p*=.030, η_p_^2^=.10. Error rates in Task 2 showed no significant results, *F*s≤2.54, *p*s≥.118.

#### Omissions

Omission rates showed no significant results in either Task 1, *F*s≤3.08, *p*s≥.086, or Task 2, *F*s≤2.24, *p*s≥.141.

### Discussion

In Experiment 2, we made the effects of Task 1 unpredictable to exclude any strategic or motivational influences, and to reduce the relevance of the action effects as a feedback signal. As expected, with unpredictable effects we no longer found any influences of compatibility in Task 1. But crucially, the influence of compatibility still showed up in Task 2, indicating that even unpredictable effects were monitored, and that the monitoring for incompatible effects took slightly longer than for compatible effects (cf., Wirth, Janczyk, & Kunde, 2018a, Experiment 5). Again, the size of the compatibility effect was not reduced without overlapping response dimensions in both tasks (even though the Bayes factor did not provide clear evidence in favor of the null hypothesis), indicating that monitoring of (spatial) action effects is rather free from feature binding influences.

For now, we can conclude that spatially incompatible effects lead to a slowdown in a subsequent task relative to compatible effects. We attribute this difference to attentional processes that we call effect monitoring, and we assume that incompatible effects take longer to monitor (Wirth, Janczyk, & Kunde, 2018a); however, feature binding processes do not seem to contribute to this slowdown to a large extent.

## Experiment 3

For the final experiment of the series, we wanted to test whether it makes a difference if R2 repeats R1 or if it repeats E1. To achieve this, we took the setup of Experiment 1 and changed the mechanics, so that responses in Task 1 no longer produced spatially compatible or incompatible effects in the same dimension as the response (vertical vs. horizontal), but so that a vertical R1 always lead to a horizontal effect and a horizontal response always lead to a vertical effect. This way, a subsequent R2 always produces a dimensional overlap with either R1 or E1, but never both, and feature binding processes can only lead to partial or full alternations (see Table 2).

**Table 2:**
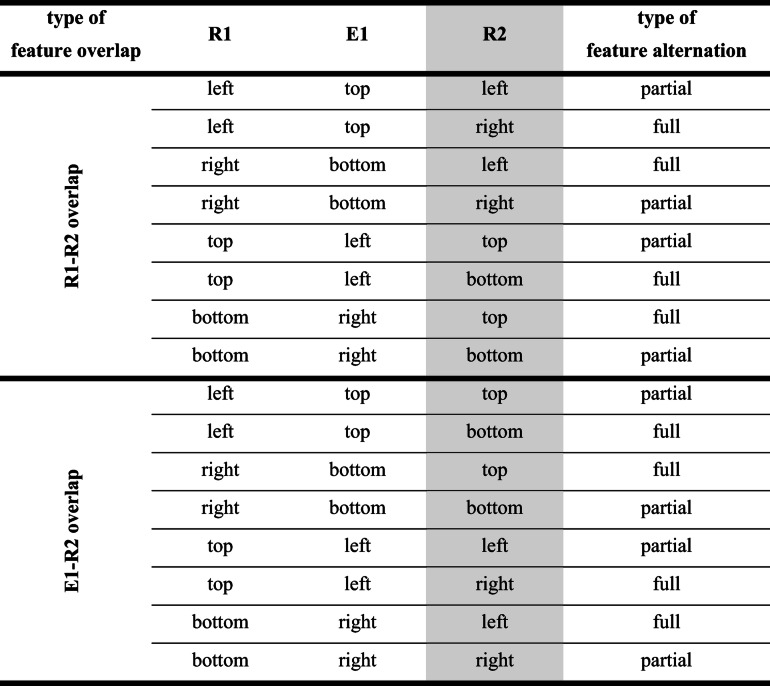
Design of Experiment 3. Horizontal R1s would produce vertical E1s, and vertical R1s would produce horizontal E1s. In this example, “left” was associated with “top,” and “right” was associated with “bottom.” Based on the R2, there could either be an overlap between R1 and R2 (upper rows) or between E1 and R2 (lower rows). Assuming feature binding processes between R1 and E1, R2 could either alternate all spatial codes of Task 1 (full alternation) or repeat either the direction of R1 (R1-R2 overlap, partial alternation) or the direction of E1 (E1-R2 overlap, partial alternation)

### Method

Forty-eight new participants were recruited (mean age 23.9 years, SD 3.7) and received monetary compensation. They fulfilled the same criteria as in Experiments 1 and 2.

Apparatus, stimuli, and procedure were exactly as in Experiment 1, with the following changes: Again, there was only one experimental session, and the R1-E1 compatibility manipulation was removed completely. Now, a horizontal R1 would produce a vertical E1 and a vertical R1 would produce a horizontal E1. One-half of the participants were instructed that “left” and “top” belonged together, as well as “right” and “bottom” (R1→E1 combinations: left→top; top→left; right→bottom; bottom→right; see Table 2), the other half was told that ”left” and “bottom” went together, as well as “right” and “top” (left→bottom; bottom→left; right→top; top→right), for counterbalancing. This mapping would stay constant for each participant during the whole experiment. Again, participants were informed before each block which response dimension would be relevant for the two tasks during the upcoming block (vertical vs. horizontal in Task 1; vertical vs. horizontal in Task 2), and they were also reminded of the R1-E1 mapping.

Participants again completed 20 blocks, five blocks per R1-R2 combination. Again, the order of the R1-R2 combinations was counterbalanced across subjects. Each block consisted of 80 trials, with 40 forced-choice trials and 40 free-choice trials in randomized order. In forced-choice trials, each combination of S1 (left vs. right connector; or top vs. bottom connector; depending on the R1 dimension of the current block) and S2 (blue vs. orange splash; or red vs. green splash; depending on the R2 dimension of the current block) was presented ten times (2 × 2 × 10), and in free-choice trials, the free-choice stimulus (left and right connectors; or top and bottom connectors; depending on the R1 dimension of the current block) was paired with each S2 20 times (1 × 2 × 20).

### Results

In free-choice trials, all four response options in Task 1 were chosen with an equal ratio (left key: 23.2%, right key 26.6%, top key: 25.5%, bottom key 23.9%, *t*s≤1.88, *p*s≥.066, *d*s≤0.27, response omissions: 1.0%). For RT analyses, we excluded trials with errors (Task 1: 6.9%, Task 2: 9.2%) and omissions (Task 1: 1.1%, Task 2: 1.1%). Outliers were removed as in the previous experiments (4.6%). Overall, 20.3% of the trials were removed.

**Fig. 5:**
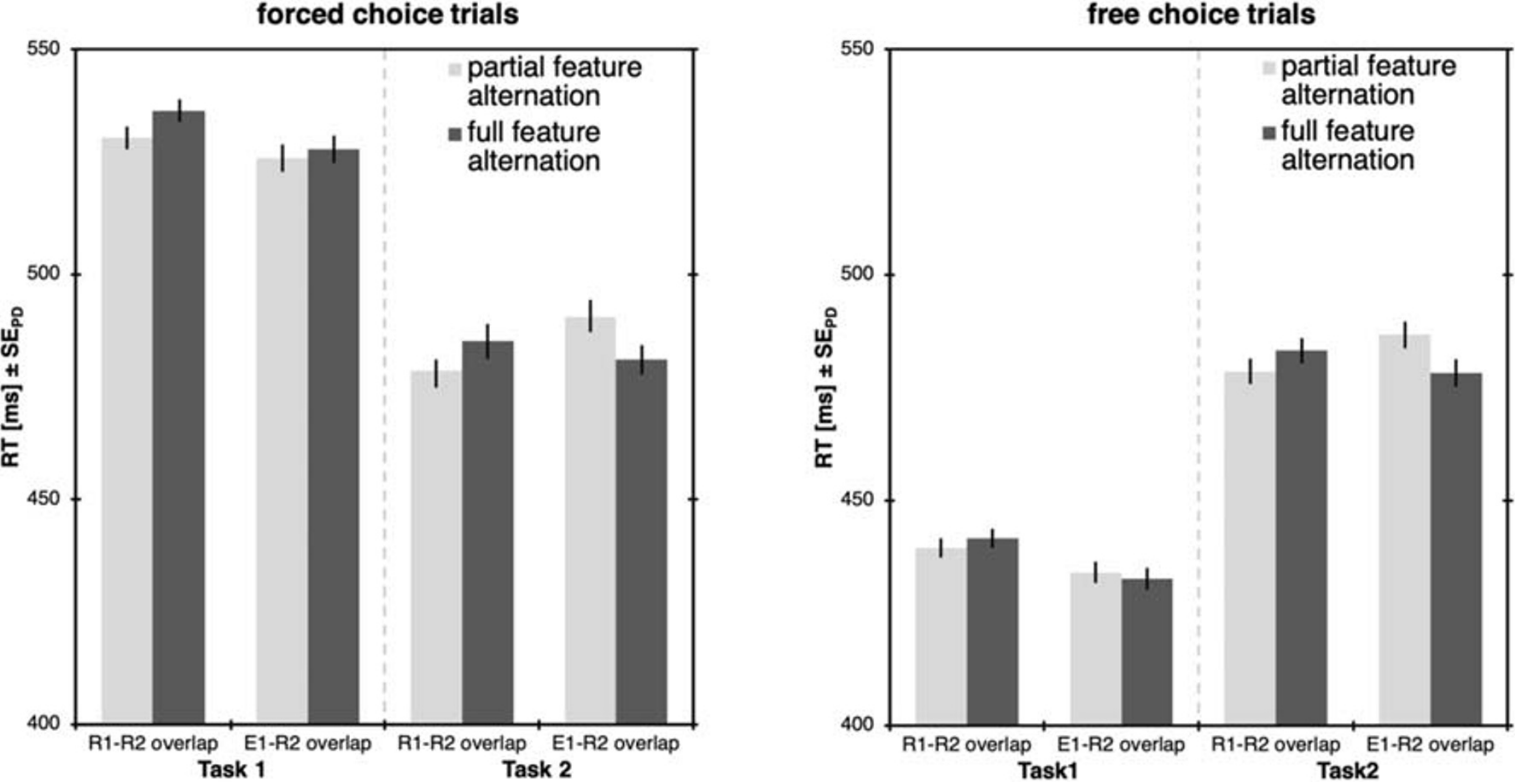
Results of Experiment 3. Response times (RTs) for forced-choice trials (**left**) and free-choice trials (**right**), separately for Task 1 and Task 2. Light bars represent partial feature alternations, dark bars represent full alternations. Error bars denote the standard error of paired differences, computed separately for each comparison of feature overlap (Pfister & Janczyk, 2013)

The remaining data were then analyzed separately depending on whether Task 1 was forced or free choice. RTs were analyzed with a 2 × 2 ANOVA with type of feature overlap (R1-R2 overlap vs. E1-R2 overlap) and type of feature alternation (partial vs. full) as within-subjects factors (see Fig. 5). In case of errors and omissions, it is difficult to assess what is actually bound into an event file in Task 1, so error rates and omissions will only be analyzed with the factor type of feature overlap.

#### Forced choice trials

##### Task 1

Task 1 produced no significant results, *F*s<1.

##### Task 2

In Task 2, none of the main effects turned significant, *F*s<1, but they combined to a significant interaction, *F*(1,47)=9.68, *p*=.003, η_p_^2^=.17, with significant RT costs for partial feature alternations relative to full alternations in case of E1-R2 overlap, *t*(47)=2.79, *p*=.008, *d*=0.40, ∆=9 ms, and a non-significant RT benefit for partial feature alternations relative to full alternations in case of R1-R2 overlap, *t*(47)=-1.65, *p*=.106, *d*=-0.24, ∆=-6 ms.

##### Errors

Error rates did not differ between overlap conditions in either Task 1, *t*<1, or Task 2, *t*(47)=1.70, *p*=.096, *d*=0.24

##### Omissions

Omission rates did not differ between overlap conditions in either task, *t*<1.

#### Free-choice trials

##### Task 1

Task 1 produced no significant results, *F*s≤1.33, *p*s≥.254.

##### Task 2

In Task 2, none of the main effects turned significant, *F*s<1, but they combined to a significant interaction, *F*(1,47)=18.86, *p*<.001, η_p_^2^=.29, with significant RT costs for partial feature alternations relative to full alternations in case of E1-R2 overlap, *t*(47)=2.89, *p*=.006, *d*=0.42, ∆=9 ms, and a non-significant RT benefit for partial feature alternations relative to full alternations in case of R1-R2 overlap, *t*(47)=-1.88, *p*=.066, *d*=-0.27, ∆=-5 ms.

##### Errors

Error rates did not differ between overlap conditions in either task, *t*s<1.

##### Omissions

Omission rates did not differ between overlap conditions in either Task 1, *t*<1, or Task 2, *t*(47)=1.08, *p*=.288, *d*=0.16.

### Discussion

In Experiment 3, we changed the mechanics of Task 1 so that horizontal responses would produce vertical effects and vice versa, so that R1 and E1 are no longer mapped in a spatially compatible or incompatible manner. With the current setup we were able to detect whether it makes a difference to repeat the R1 or E1 dimension in Task 2.^5^

For those cases in which the R2 dimension overlaps with the E1 dimension, we found that full alternations were consistently faster than partial alternations in both forced and free-choice trials, again showing that partial feature repetition slows down responding. Binding of R-E features in a preceding task thus has an impact on the generation of a response in a subsequent task.

However, those trials in which the response dimensions of both tasks overlap paint a somewhat different picture. Differences between full and partial alternations are not statistically significant here, but if taken seriously, they go in the opposite direction to what one would usually expect, with full alternations taking longer than partial alternations. Again, we do not want to read too much into this, but for the interpretation, keep in mind that here, partial alternations basically mean response repetitions (even though different hands were used, but R1 and R2 required the same spatial response code), which have been shown to come with their own advantages (e.g., Bertelson, 1965). This benefit of response repetitions was stronger in the free-choice trials, but in no case did they meet the conventional levels of significance.

Taken together, these results show that feature binding can contribute to effect monitoring, and that binding between R1 and E1 can produce both benefits and costs in Task 2, depending on whether and which of the codes has to be repeated.

## General discussion

Monitoring the effects of our actions can interfere with concurrent cognitive processes of another task (Welford, 1952). Thereby, it can delay the generation of a motor action in case of high monitoring demands (Wirth, Janczyk, & Kunde, 2018a). Here, we asked whether feature binding processes (especially when repeating some but not all previously bound features) are the main driver of these response delays, or whether feature binding costs are relatively independent of monitoring costs.

We observed that feature binding plays a role in the experiments that we present here. In Experiments 1 and 2, performance was overall impaired in Task 2 if there was a dimensional overlap between the two tasks relative to when there was not. With overlap in the response dimension (see Fig. 2), there is the possibility of producing sequences in which all, some, or none of the R1-E1 features repeat in Task 2 (see Table 1), allowing for the contribution of binding costs, and also confounding the influence of spatial compatibility. However, when there is no dimensional overlap in the responses of the two tasks, possible feature binding costs cannot manifest in Task 2 performance, and we see overall faster and more accurate responses in Task 2 when feature binding contributions are impossible. Still, this absence of feature binding did not mitigate the influence of spatial R1-E1 (in)compatibility on Task 2, which did not become smaller when feature binding contributions were removed. Thus, we can conclude that while feature binding contributes to participants’ overall performance, it does not drive the increased costs of monitoring incompatible as compared to compatible action effects by itself.

A second index for feature binding processes could be found in Experiment 3, in which we omitted the manipulation of spatial compatibility. In this setup, we found that if a response requires the spatial dimension that was previously bound as an effect code, this response is administered significantly slower than when there is no overlap between effect and response codes. This result is consistent with previous research (e.g., Moeller, Pfister, Kunde, & Frings, 2016, 2019). Thus, we can conclude that, in principle, specific R1-E1 episodes (that are likely built up during monitoring) can also delay performance in a subsequent task and thereby contribute to the overall monitoring costs.

Taken together, it seems as if feature binding processes are not the only source for monitoring costs, but that contributions due to the incompatibility of actions and effects represent a separate source of interference. Possibly, incompatible action effects, even when they are expected in the experimental context, still violate pre-experimentally established experiences of usually producing spatially compatible effects in the environment (e.g., Wirth, Janczyk, & Kunde, 2018a). This violation of expectations might then prompt a general *stop* signal in the cognitive system, which might drive the delayed responding after incompatible effects (Wessel & Aron, 2013).

As an alternative to assuming that feature binding processes and spatial incompatibility contribute independently to the performance of the subsequent task, one might also assume either that (a) incompatible features are bound less strongly into an episode, or that (b) episodes in which incompatible features are bound are not retrieved as automatically. Speculatively, it would make sense to assume that features should not, or only to a lesser degree, retrieve episodes that were problematic, negative, triggered conflict, or that may have led to an error (for related ideas on errors, see Wiswede, Rothermund, & Frings, 2013; on valence, see Waszak & Pholulamdeth, 2009). Assuming that effect incompatibility hinders either the generation or the retrieval of an event file, we would also expect slower responses after an incompatible effect compared to after a compatible effect. If that were the case, these “incompatibility costs” would then not constitute partial alternation costs as described so far (Table 1); rather the difference between responses after compatible and after incompatible effects would only be driven by faster full repetitions and full alternations, and therefore should be better described as a “compatibility benefit”. Hence, for future research it might be interesting to study whether spatially incompatible or otherwise uncommonly associated feature combinations are generally less likely to be bound and/or retrieved as compared to spatially compatible or otherwise common feature ensembles (but see Pfister, Frings, & Moeller, 2019, on this topic).

At any rate, we show here that removing the possibility of feature binding did not eliminate the increased costs of monitoring incompatible as compared to compatible action effects. This might be because partial feature repetitions and monitoring of spatial incompatibility represent separate sources of processing delays, or because conflict-associated feature episodes are bound and/or retrieved differently from less problematic, conflict-laden episodes.

## Footnotes

1 One may argue that the slowdown of responding in Task 2 after an incompatible action effect in Task 1 resembles sequential conflict adaptation, thus a reduction of S-R compatibility effects after incompatible S-R episodes (Gratton, Coles, & Dochins, 1992). Yet, there are a couple of differences between sequential conflict adaptation and the slowdown of responding after incompatible action effects we studied here. First, there was no compatibility manipulation whatsoever in Task 2, making it impossible for conflict adaptation to manifest in the first place. Second, we did not manipulate S-R compatibility but R-E compatibility. Third, even if we assume that incompatible R-E episodes prompt similar adaptations to incompatible S-R episodes, there is typically a slowdown for compatible after incompatible trials, but a benefit for incompatible after incompatible trials, hence no overall slowdown after incompatible events. Finally, sequential conflict adaptation has itself been explained by partial feature repetition costs (Hommel, Vu, & Proctor, 2002), as it is strongly reduced when feature repetitions are removed (Mayr, Awh, & Laurey, 2003). In contrast, here we will show that the RT increase after incompatible rather than compatible action effects remain even when feature repetitions are removed. For these reasons we find it unlikely that the mechanisms causing sequential conflict adaptation play a strong role in the slowdown of responding after experience an incompatible action effect that we studied here.

2 As the current experiments revolve around the spatial relation between R1, E1, and R2, we will focus on the spatial features left, right, top, and bottom. These are not to be confused with the spatial dimensions, horizontal and vertical, on which these features can vary.

3 Based on the comments of an anonymous reviewer, we specifically compared full alternations against partial alternations in the dimensional overlap condition (excluding all full repetitions, see Table 1). Even with full repetitions removed, we found faster responses for full alternations compared to partial alternations for both forced-choice, *t*(47)=3.83, *p*<.001, *d*=0.55, ∆=25 ms, and free-choice, *t*(47)=3.59, *p*=.001, *d*=0.52, ∆=25 ms, trials.

4 Based on the comments of an anonymous reviewer, we specifically compared full alternations against partial alternations in the dimensional overlap condition (excluding all full repetitions, see Table 1). Even with full repetitions removed, we found faster responses for full alternations compared to partial alternations, *t*(47)=4.84, *p*<.001, *d*=0.70, ∆=8 ms.

5 We also performed a similar analysis on a subset of the data of Experiments 1 and 2. Looking at only the incompatible trials of those blocks in which both response dimensions overlapped, we also find cases in which R2 only overlaps with R1 (e.g., R1-E1: left→right; R2: left) and others in which R2 only overlaps with E1 (e.g., left→right; right). Within these cases, we cannot differentiate between different types of feature binding (rather, they are confounded, R1R2 repetitions are automatically E1R2 alternations and vice versa), but their direct comparison might give us an estimation of whether there is an overall benefit for repeating either R1 or E1 over the other. We do not find any effects in Experiment 1, *t*s≤1.61, *p*s≥.112, but we find a systematic benefit for R1 repetitions (over E1 repetition) in Experiment 2, *t*s≥2.78, *p*s≤.008, *d*s≥0.40, ∆≥10 ms.
